# Supplementary material for: Digital nanoreactors to control absolute stoichiometry and spatiotemporal behavior of DNA receptors within lipid bilayers
Source: Nat Commun. 2023 Mar 20;14:1532. doi: 10.1038/s41467-023-36996-x (PMC10027858; doi:10.1038/s41467-023-36996-x)
Supplement: Supplementary file 1 — Supplementary Information [file 41467_2023_36996_MOESM1_ESM.pdf]

Supplementary information

Supplementary Note 1: Analysing different fractions

We performed initial experiments to determine which fractions contain DOL among the different fractions collected after isopycnic purification step (Step D Figure 1a and Supplementary Figure 1d). We confirmed this by transmission electron microscopy (TEM) images and fluorescence measurements using a plate reader instrument on different fractions for DOL^{1A1B} case (Supplementary Figure 4). TEM data suggested that fractions 1 and 2 have only free liposomes, and the count of free liposomes is higher in fraction 1 than fraction 2. Most of the DOL were found to be present in fractions 3-5 and rarely observed beyond fraction 6. TEM images for DOL clearly show a DNA ring structure around a spherical blob.

To corroborate TEM observations we performed plate reader experiments. Ideally, assembly and purification steps explained in Figure 1a should result in a DOL platform with the tethered receptors (mainly found in fractions 3-5 as observed in TEM) without any free diffusing receptors either in the solution or on the DOL surface. It is more likely that free cholesterol receptors, if there are any, should anchor in the membrane surface of free liposomes (fractions 1 and 2) or DOL lipid bilayer (fractions 3-5) rather than diffusing in the bulk solution. It is important to note that free receptors are active and both the receptors can cooperatively displace the top quencher strand from the reporter complex which could result in undesired fluorescence signal. Keeping these factors in mind we tested the logic circuit in all the collected fractions (up to 10). As shown in Supplementary Figure 4, we started by mixing each fraction with the reporter complex (final concentration 4.7 nM) and for initial 7 h we did not observe any major fluorescence signal in any fraction. If there were any free receptors, in any fraction, then it would have resulted in a rise in signal over the initial 7 h. After 7 h, both release strands were added which released both the receptors (thus active) and their cooperative hybridisation with reporter complex resulted in a sudden rise in fluorescence signal. The saturation in a signal (amount of reporter consumed) is proportional to the receptor concentration; thus the DOL concentration which contains the receptors. Both the TEM data as well as fluorescence data correlate very well. TEM data suggested that fractions 3 and 5 have lower DOL concentration (based on visual counts) than fraction 4. Similarly, the fluorescence signal obtained from fraction 3 and 5 is lower than the fraction 4. Based on this data, for our other experiments we decided to combine fractions 3 and 4 to have more volume in hand to perform repeats and other controls. In contrast, the highest concentration of DOL in dimer_DOL^{1A1B} case (Supplementary Figure 7) was found to be in fraction 5 (Supplementary Figure 8) instead of fraction 4 as in the case of DOL^{1A1B}, which is expected because dimer_DOL^{1A1B} has two rings dimerised together.

For some experiments fractions 3 and 4 were pooled to have more volume for analyses. In Figure 2d, the kinetics curve for DOL^{1A1B} is shown for the pooled fractions 3 and 4, which has higher fluorescence intensity, thus higher DOL concentration, than fraction 5 (compared in Supplementary Figure 5a). On the other hand, in the case of DOL^{2A2B} (Figure 2d) the fluorescence kinetics curve is shown for fraction 5, but the pooled fractions (3+4) consumed the reporter complex completely

(Supplementary Figure 5a) which implies that total receptor concentration in pooled fractions (3+4) was at least high as the reporter concentration (see related discussion in Supplementary Notes 3 and 4). Thus, the fluorescence curve for combined fraction (3+4) in DOL^{2A2B} case was not used to perform additional analyses (e.g. measuring concentration or deriving rate constants).

Supplementary Note 2: **Miscellaneous notes**

a) Our purification step (Step A, Figure 1a) removes most of the staples, including linkers, and further the purified fractions (~ 97% origami band intensity, ~ 3% traces staples band intensity, Supplementary Figure 1b) were pooled and concentrated using Amicon 30 kDa centrifuge filters (see Methods) which should additionally get rid of traces of staples or linkers. Note that ~3% band intensity is for all the staples (~200 in number) and the contribution from linker strands (2-4 in number) would be almost negligible (if contribution is equally weighed among all strands then it would be < 0.02%). If we assume, which is less likely, the traces of linker strands still remain around after Step A purification and the filter concentration step (Figure 1a) then the free linkers may hybridise with the receptors in solution forming traces of 'protected' receptors at Step B (Figure 1a). If we further assume that traces of protected receptors were not removed at all, which is again unlikely, in further next steps (Step B purification) then at least one can say all of the signal (curve kinetics) that we see for DOL after the release strand is added is originating from > 99% of tethered receptors and rest is the undesired reaction coming from protected receptors on the DOL surface.

b) Reported[1] (and references there in) studies suggest that Mg^{+2} can cause binding of unmodified DNA on the GUVs in their gel phase (made with DPPC and below melting temperature) due to bridging effect of the divalent ion between lipid head groups and DNA backbone. But in the liquid phase Mg^{+2} does not cause DNA (unmodified) binding on the GUV surface. MD simulations[1] suggest Mg^{+2} has more affinity for the lipids in gel phase because of higher coordination number with head groups compared to the lipids in liquid phase. However, in the liquid phase cholesterol modified DNA was able to bind the GUV only in the presence of Mg^{+2} (or other cations) where cations play a different role of screening the charge repulsion between DNA-lipid (not the bridging effect) and DNA remains bound to the lipid surface because of cholesterol anchors.[1]

In our case we have used a mixture of DOPC and DOPS which have melting temperatures (Tm) '-17 °C' and '-11 °C' respectively. It is expected that our DOL liposomes are in the liquid phase (plate reader experiments conducted at 25 °C) and, as discussed above, the DNA receptors are thus anchored in lipids due to cholesterol terminals instead of nonspecific DNA binding on the lipid surface due to Mg^{+2} bridging effect (possible in gel phase). This is supported by a reported case[1] in which they did not observe DNA (unmodified) binding with POPC GUVs at room temperature (POPC has a low Tm '-2 °C' as in our case of DOPC and DOPS). Also to mention, our buffer contains monovalent ion (100 mM KCl) which is known to suppress the bridging ability of divalent ions but instead could help charge screening; both effects are well reported.[1] To summarize this, our receptors are expected to be anchored in the lipids with the cholesterol tags instead of ion mediated bridging

effect. Additionally, we have DOPS 25% and PEG2000-PE 5% both as negative charged lipids present in our liposome. High negative charge density could further also prevent sticking of DNA on the lipid surface due to repulsion. We expect that all these factors have minimal effect on the DNA dimerization process, thus we assume, also in our simulation models, sticky ends of DNA (receptor dimerization domains) to be free and not lying flat on the lipid surface.

Supplementary Note 3: **Concentration from saturation end points**

For the cases (main Figures 2d and 3b, Supplementary Figures 5a, 6, and 8) where the reporter complex is not fully consumed, the saturation end points could be used to determine the DOL concentration. This requires measurement of maximum available fluorescence to know the amount of unreacted reporter, which we performed in all the cases by adding excess of stimulant strands (non-cholesterol versions of anchor_A and anchor_B). Before explaining how we used this method to determine DOL concentration, it is important to note that adding excess of a reporter complex does not occupy/block free individual receptors (partial displacement of BHQ top strand by free single receptor; A^i and B^i states shown in Supplementary Figure 2). To justify this, as shown in Supplementary Figure 6, when the same DOL^{1A1B} sample is mixed with two different reporter concentrations, 4.7 nM and 14 nM (c.a. 3 times higher) in both the cases the kinetic curves end almost at the same saturation point after the receptors are released to react with the reporter; implying that same amount of reporter complex is consumed. If excess reporter were to occupy both the receptor types individually, in the form of favorable stable intermediate states (A^i and B^i), then the end points (reporter consumed by receptors) reached using two different reporter concentrations would not have been the same leaving some receptors incapable to react cooperatively (saturation level in case of 14 nM reporter concentration expected to be lower in such a scenario). Additionally, the final end product, a ternary complex showing fluorescence, is thermodynamically more favourable than the intermediate states (A^i and B^i), which makes the forward process almost irreversible. Overall, this implies that the receptor and reporter molecules would be consumed irreversibly in the form of a final ternary complex only (without any stable intermediate states) and yielding a fluorescence signal. In our experiments the release strands are added in excess (100 nM), which makes TMSD receptor release almost complete and ensures that the receptors on every DOL are available and active to react with reporter complex present in solution. To summarize this, for the cases where receptor concentration is lower than reporter concentration we used saturation end points to determine the DOL concentration.

Furthermore, we can counter the argument that we do not know whether release strands were in excess or not because we do not know DOL concentration in any fraction (each 50 μL), by pointing out that the release strands (100 nM) are indeed in excess of the starting raw material itself before purification (ring at 15 nM, 150 μL at Step D, Figure 1a). Further supporting this point is the fact that we used a known concentration, 5 nM of a purified ring tethered with one Receptor_A and one Receptor_B (obtained after Step B, Figure 1a), and then quantified the concentration of each receptor independently by releasing one receptor at a time (Figure 3b; orange and green curves). This data showed that the two receptors were at almost equal

concentrations and their individual concentrations were almost equal to the ring as well (5 nM).

Therefore, considering the discussion above, we can assume that the saturation end point is simply not the measure of concentration of species in equilibrium but is an indication that the reaction goes to completion consuming almost all the available receptors on each DOL. This is the rationale we employed in using saturation end points as the measure of DOL concentration as shown in Figure 2d; [DOL^{1A1B}] is ~2.4 nM, [DOL^{2A2B}] is ~1.4 nM and [dimer_ DOL^{1A1B}] is ~1.2 nM. Note that in the case of DOL^{2A2B}, the DOL platform concentration (for fraction 5, Figure 2d) would be half of the receptor concentration (saturation point), where receptor concentration is equal to the reporter consumed. Concentration of DOL^{2A2B} in a combined fraction (3+4) cannot be evaluated from the curve saturation end points as all the reporter molecules were consumed (Supplementary Figure 5a); at 36 h adding excess of stimulant strands did not show any further spike in the signal but receptor concentration is at least 4.7 nM. Nonetheless, we indirectly estimated DOL^{2A2B} concentration in the combined (3+4) fraction using TEM data (Supplementary Note 4) and found it to be c.a. 2.98 ± 0.53 nM, thus making receptor concentration c.a. 6 nM. Thus, these results are in good agreement with the plate reader observation that the combined (3+4) fraction consumed all the reporter molecules (at 4.7 nM).

Supplementary Note 4: **Concentration from TEM**

As discussed in Supplementary Note 3 above the kinetic curves, Figure 2d, show that the reporter complex is not fully consumed in these cases and thus saturation end points could be used as a measure of DOL concentration. We also collected TEM images for DOL^{1A1B} samples which allowed us to correlate the number of counts of DOL (done manually) with the concentration obtained from the plate reader experiment. Linear regression fitting of concentration (from plate reader) of DOL^{1A1B} vs. their averaged TEM counts (taken from at least 4 TEM images for DOL^{1A1B} case from two different fractions) provided a general relationship between counts and concentration (Supplementary Figure 5b). This allowed us to calculate concentration of pooled fraction (3+4) in case of DOL^{2A2B} to be ~2.98 nM (green curve Supplementary Figure 5a, where all the reporter complex was consumed) from TEM data (averaged manual counts from 7 images of pooled fraction '3+4' DOL^{2A2B} sample). Similarly, for inter-DOL cases (concentrations and plots in Figure 2e), which did not reach saturation, their concentrations were calculated using the same method described here (averaged manual counts from 5 TEM images in $interDOL^{1A1B}$ case and from 4 TEM images in $interDOL^{2A2B}$ case). Calculated concentrations for interDOL cases are: ~ 1.26 nM $interDOL^{1A1B}$ case and ~ 1.03 nM $interDOL^{2A2B}$ case.

Supplementary Note 5: **Dimer DOL**

Here a slightly different type of platform is discussed. As a recall, the main motive to develop DOL platform is to start with a configuration where monomeric forms of the molecular species in question (synthetic receptors or proteins) are well separated before assaying their interaction. The DOL platform discussed in the main paper (Figure 2a and 2b) would be suitable to assay chemically induced protein interaction, for example the homo- or hetero- dimerization of proteins in the presence of a ligand

(drug, ion, peptide, soluble protein etc.) found in many protein pairs.[2] Considering our protocol step where we incubate, in a single pot, DNA origami ring with both the receptors (Step B, Figure 1a) it would not be suitable for proteins which could possibly form oligomers without requiring any ligand (proteins with constitutive interactions). For a chemical induced dimerization process this would not be an issue because at a specific tethering site there will be only one type of monomer present. For constitutive proteins this protocol could be problematic because there could be already an oligomeric form of protein complex attached at a specific tethering site. Though one could potentially test different conditions (e.g. detergents, salt concentrations, inhibitors etc. which can be later dialysed out at Step D, Figure 1a) to prevent oligomerisation at the incubation step but to overcome this we devised a slightly different strategy. We incubated separately each type of receptor with a DNA ring having a specific complementary linker and purified them individually (Supplementary Figure 7). Later, two DNA rings (each with only one type of receptor; Receptor_A or Receptor_B) were dimerised with the help of 16 complementary sticky staple end extensions on one ring to the scaffold of second ring. To assemble a liposome inside the dimerised rings (dimer_DOL^{1A1B}) we further followed the same protocol employed for DOL platforms. Similarly, receptors were released in dimer_DOL^{1A1B} case and the kinetic curve for fraction 6 is shown Figure 2d (green curve). Supplementary Figure 8 shows the kinetic curves for different fractions using reporter complex at two different concentrations. Though for our dimer_DOL^{1A1B} we still use chemically induced dimerization (where DNA receptors dimerise in the presence of reporter) we hope that similar strategy could be used for constitutive proteins as well.

Supplementary Note 6: **Kinetics simulation**

The reactions in this work were simulated using mass-action kinetics. Each reaction was modelled as a differential equation which was then solved by using a CRN Simulator Package.[3]

There are several reaction steps, listed below.

1) $\text{release\_A} + \text{Receptor\_A(bound)} \xrightarrow{K_{eff}} \text{Receptor\_A(free)} + \text{release\_A:linker\_A}$

2) $\text{release\_B} + \text{Receptor\_B(bound)} \xrightarrow{K_{eff}} \text{Receptor\_B(free)} + \text{release\_B:linker\_B}$

3) $\text{Receptor\_A(free)} + \text{Reporter} \underset{K_b}{\overset{K_f}{\rightleftarrows}} \text{Receptor\_A:Reporter}$

4) $\text{Receptor\_B(free)} + \text{Reporter} \underset{K_b}{\overset{K_f}{\rightleftarrows}} \text{Receptor\_B:Reporter}$

5) $\text{Receptor\_A:Reporter} + \text{Receptor\_B(free)} \xrightarrow{x.K_{eff2}} \text{TernaryComplex} + \text{Top strand}$

6) $\text{Receptor\_B:Reporter} + \text{Receptor\_A(free)} \xrightarrow{x.K_{eff2}} \text{TernaryComplex} + \text{Top strand}$

In reactions (1) and (2), release_A and release_B strands bind to their respective receptors in order to free them from the linker strands. The strand displacement

reaction is irreversible and described by rate K_{eff}, which is the effective rate of strand displacement. In reactions (3) and (4), the free receptors bind to the reporter. The forward rate, K_f, is the rate of hybridization of the toehold; the backward rate, K_b, is the rate of dissociation, which is determined by the formula[4] 10^{6-L} $M^{-1}s^{-1}$, where L is the toehold length, in this case L = 5. In reactions (5) and (6), the receptor:reporter complex binds to the other receptor and does strand displacement at the rate K_{eff2}, which describes the rate of cooperative strand displacement. This rate was determined using simulations of the reaction system in solution (using strands without cholesterol modifications, Figure 2f). The factor x is used to describe the speed-up in the reaction rate between the solution case and the DOL case, which in turn gives us insight into the relative concentrations of the receptors when they are on the DOL. This becomes clear when we look at the differential equations for that set of reactions:

$$(7)\quad \frac{d[\text{TernaryComplex}]}{dt} = x * K_{eff2}\ [\text{Receptor\_A:Reporter}]\ [\text{Receptor\_B(free)}]$$

The rates used are[4,5]:
$K_{eff} = 2*10^5\ M^{-1}s^{-1}$ [ref[4]]
$K_f = 2*10^6\ M^{-1}s^{-1}$ [ref[5]]
$K_b = 10\ s^{-1}$
$K_{eff2} = 7.6*10^4\ M^{-1}s^{-1}$

Experimental curve fittings based on this model are shown in Supplementary Figure 10.

Supplementary Note 7: **DNA ring origami design and staple sequences**

DNA origami ring design (caDNAno json file) and staple sequences (including linkers for receptors) are provided as Supplementary Software and Supplementary Data files respectively. Nanocage_BASIC.json is a general design and the staple sequences for a specific design can be found with their helix and residue numbers (caDNAno format) in .xlsx file. For dimerization of rings in dimer_DOL^{1A1B} case there are 16 staples extended (labelled as dimer-linker in Ring2_for_dimer1A1B_sequence) with small sticky ends (4 nucleotides) from one ring which hybridize with the scaffold of the second ring (some staples from second ring are thus kept a bit shorter).

In all cases handle sequence for cholesterol-antihandle (extension from staples):
AAATTATCTACCACAACTCAC
In all cases antihandle sequence with cholesterol (HPLC purified):
/5Chol-TEG/GTGAGTTGTGGTAGATAATTT

Supplementary Table 1: Domain decomposition of DNA receptors (see Figure 1). Domain names, their sequences (5'—3') and their roles are summarised. Colour codes used here correspond to the same coloured domains shown in Figure 1. Source data are provided as a Source Data file.

Domain	Sequence (nucleotide length)	Role
Receptor_A complex (anchor_A, anchor_A*)		
A* (anchor_A*)	GTTTGAGTTGAGTGGGAAAG/3CholTEG/ (20)	Cholesterol anchor
A (anchor_A)	/5Chol-TEG/CTTTCCCACTCAACTCAAAC (20)	Cholesterol anchor
P1 (anchor_A)	CA (2)	linker_A tethering, unpaired hinge in ternary complex
X2 (anchor_A)	ACACCATTTACCCAC (15)	linker_A tethering, reporter toehold binding
X1 (anchor_A)	ATTCAAATCC (10)	Cooperative hybridisation (along with X2, Y1Y2)
linker_A (extension of staple strand connected via TTTT spacer)		
X2*	GTGGGTAAATGGTGT (15)	Receptor_A complex tethering
P1*	TG (2)	Receptor_A complex tethering
T*	AGATG (5)	Toehold for release_A
Receptor_B complex (anchor_B, anchor_B*)		
B* (anchor_B*)	/5Chol-TEG/GTTGGTAATGGAATGGGAAG (20)	Cholesterol anchor
Y1 (anchor_B)	CACAATACAC (10)	Cooperative hybridisation (along with Y2, X1X2)
Y2 (anchor_B)	CCTACACATACATCA (15)	linker_B tethering, reporter toehold binding
P2 (anchor_B)	AC (2)	linker_B tethering, unpaired hinge in ternary complex
B (anchor_B)	CTTCCCATTCCATTACCAAC/3CholTEG/ (20)	Cholesterol anchor
linker_B (extension of staple strand connected via TTTT spacer)		
S*	GTGGA (5)	Toehold for release_B
P2*	GT (2)	Receptor_B complex tethering
Y2*	TGATGTATGTGTAGG (15)	Receptor_B complex tethering
Releasing strands		
T (release_A)	CATCT (5)	linker_A toehold binding and strand displacement
P1 (release_A)	CA (2)	
X2 (release_A)	ACACCATTTACCCAC (15)	
Y2 (release_B)	CCTACACATACATCA (15)	linker_B toehold binding and strand displacement
P2 (release_B)	AC (2)	
S (release_B)	TCCAC (5)	
Reporter complex		
x2.X1.Y1.y2 (Top strand with quencher)	attacccac.ATTCAAATCC./iBHQ-1dT/.CACAATACAC.cctacacata (10.10.1.10.10) iBHQ-1dT is black hole quencher, internal modification on T	
Y2*. Y1*. X1*. X2* (Bottom strand with fluorophore)	TGATGTATGTGTAGG.GTGTATTGTG. /iFluorT/.GGATTTGAAT.GTGGGTAAATGGTGT (15.10.1.10.15) iFluorT is fluorescein, internal modification on T	

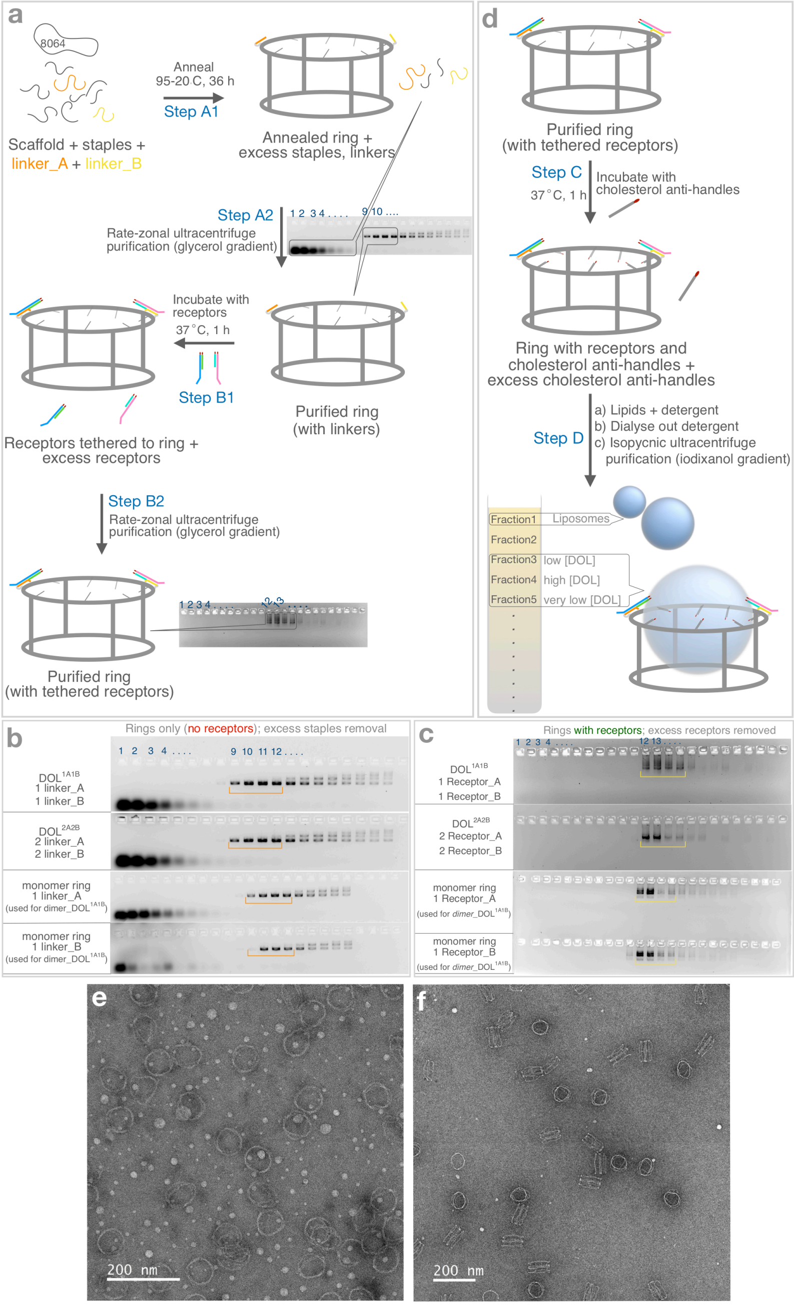

Supplementary Figure 1: DOL platform assembly and purification. Steps in Figure 1a are elaborated here for different DOL cases. **(a)** After annealing (Step A1) excess staple strands were removed (Step A2) by rate-zonal ultracentrifuge purification. Different fractions so collected were run through 1.5% agarose gel shown in **b**. Fractions containing (e.g., 9-12 shown in **b**) the purified rings were pooled and concentrated. After incubation with receptors (Step B1) excess receptors were removed by rate-zonal ultracentrifuge purification and corresponding agarose gels are shown in **c**. Fractions containing (e.g., 12-14 shown in **c**) the purified rings with tethered receptors were pooled and concentrated. **(d)** Elaborated Steps C and D in Figure 1 are shown here. Last step is shown to give an idea about the distribution of liposome and DOL in the tube after isopycnic separation. **(e)** and **(f)** show TEM images for DNA origami rings used for DOL and dimer_DOL cases respectively. White blobs seen in **e** are due to detergent (micelles) in the sample, which we added to prevent aggregation by the hydrophobic receptors tethered to the rings. For **b**, **c**, **e**, **f** similar agarose gels/TEM images were obtained in three different experiments (different day and starting materials). DOL; DNA origami liposome. DNA domains shown in different colours in **a** and **d** have a specific role as explained in Supplementary Table 1. Source data showing uncropped gels provided as a Source Data file.

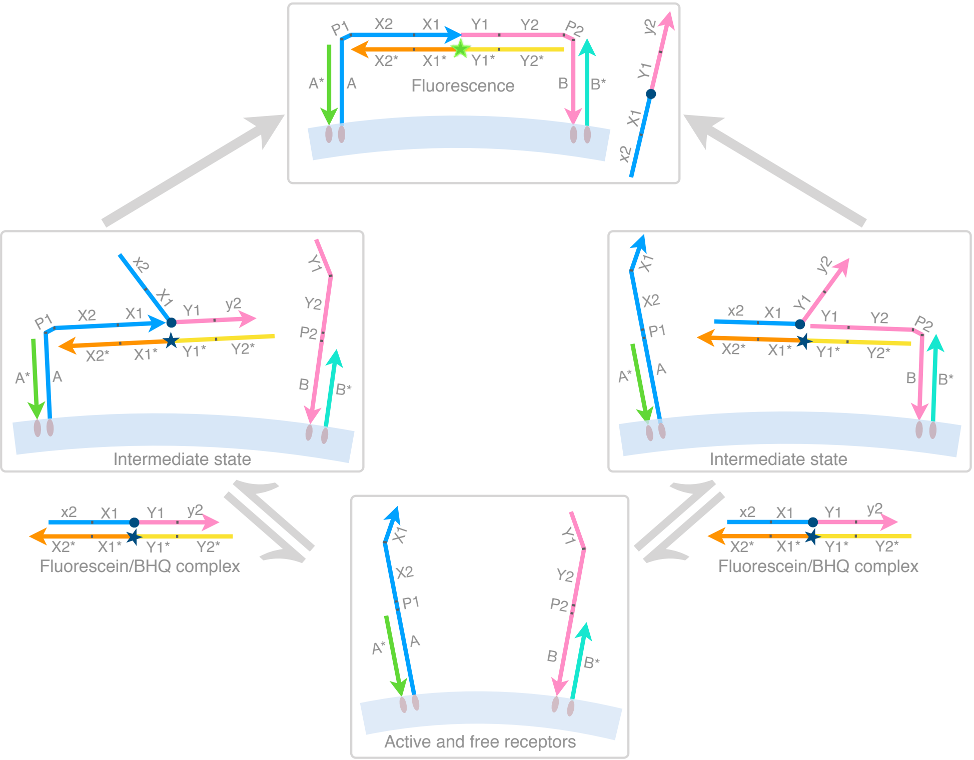

Supplementary Figure 2: Intermediate states leading to ternary complex. Possible intermediate states are shown here for the DNA circuit in Figure 1b. Lower case domains are partially complementary to their upper-case counterparts, e.g. x2 is a shortened version of X2 and is only partially complementary to X2*. A^i and B^i; intermediate states for Receptor_A and Receptor_B respectively, BHQ; black hole quencher. DNA domains shown in different colours have a specific role as explained in Supplementary Table 1.

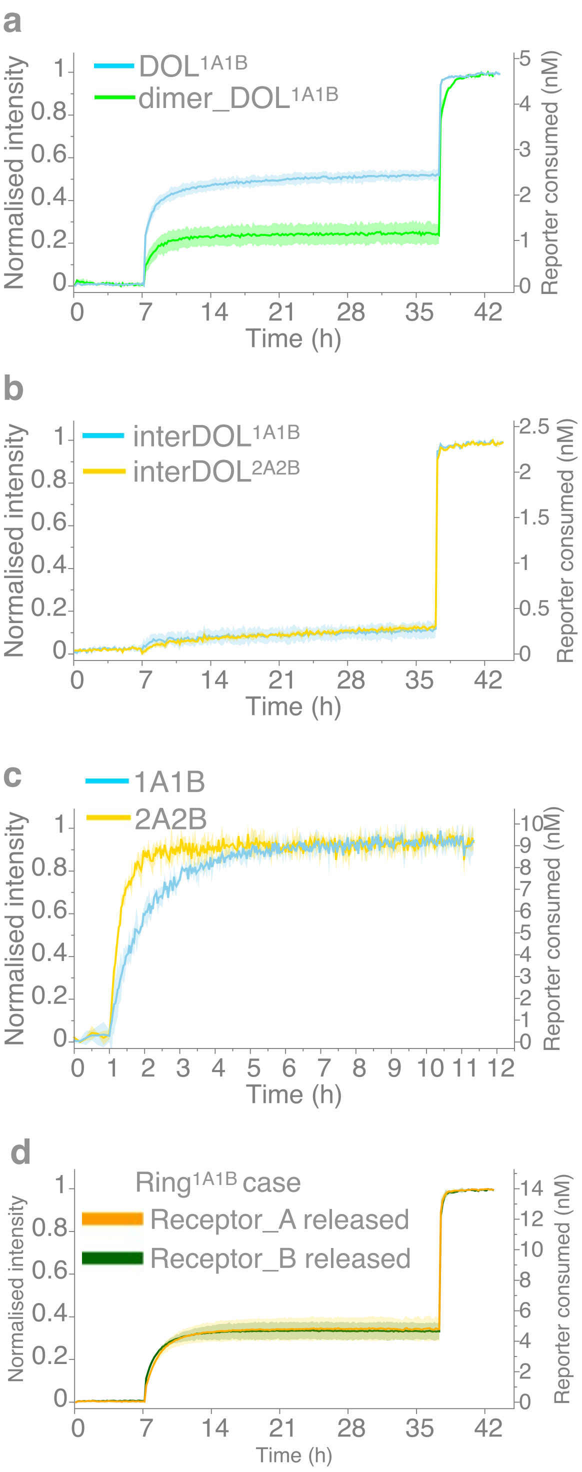

Supplementary Figure 3: Standard deviations in DOL and ring cases. Averaged kinetic curves (dark colour) for the plate reader experiments shown in main Figure 2d-f and in main Figure 3 are shown here along with their ± 2 standard deviations (corresponding transparent colour) for (**a**) DOL^{1A1B} and $\mathrm{dimer\_DOL}^{1A1B}$, (**b**) interDOL cases, (**c**) receptors in solution cases, and (**d**) receptors attached on ring. See more details in corresponding main Figure 2d-f and Figure 3 (same colour codes and abbreviations used in **a**-**d**). Source data are provided as a Source Data file.

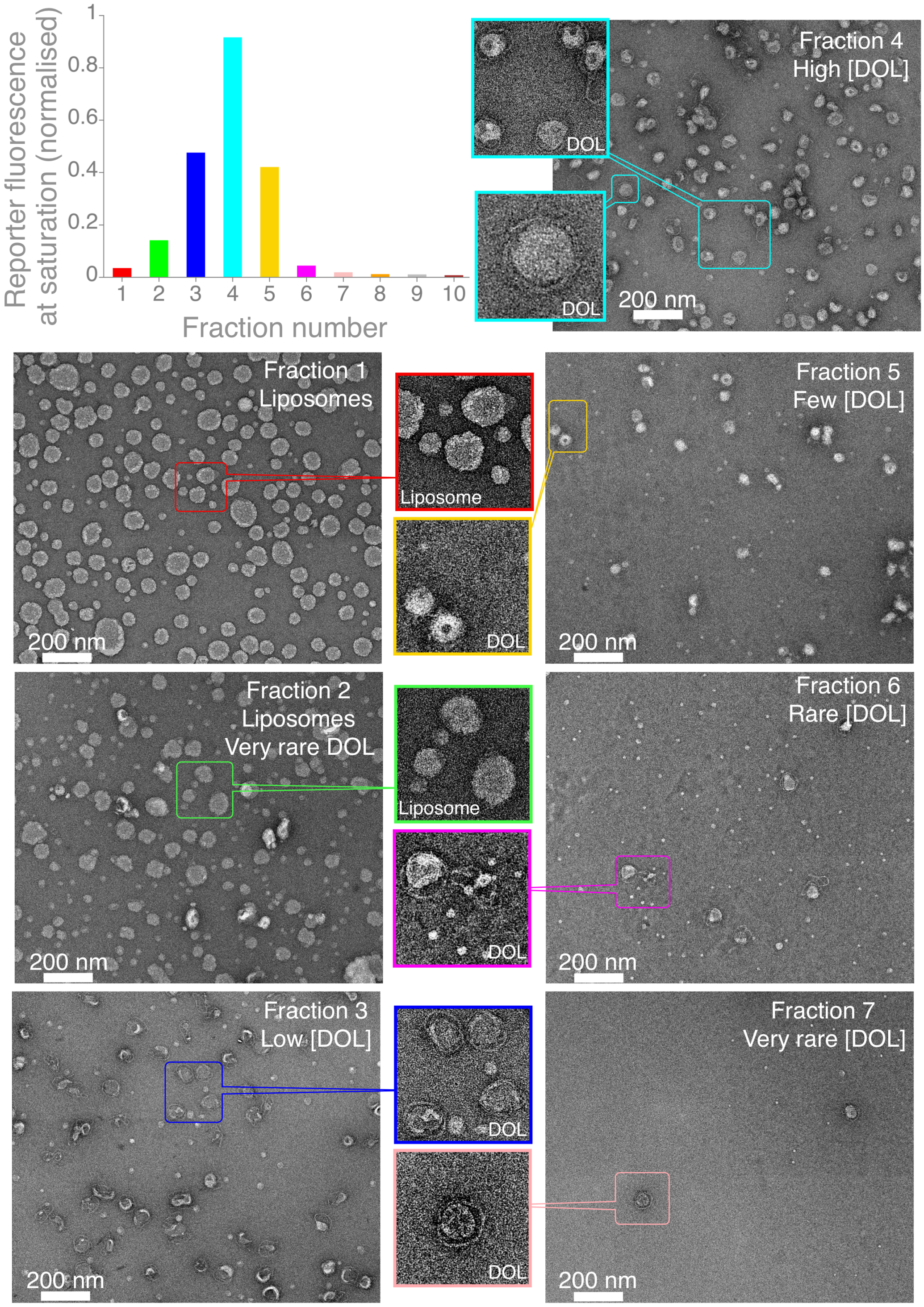

Supplementary Figure 4: Analyzing different fractions for DOL^{1A1B}. Different fractions collected after isopycnic DOL purification (see Supplementary Note 1 and Supplementary Figure 1d) were analyzed with plate reader experiment set up similar to Figure 2d. Normalized saturation for each fraction (1-10) is shown as a bar plot and TEM images are shown for fractions 1-7. Similar plate reader data/TEM images were obtained in two different experiments (different day and starting materials). For ease in comparison, coloured edges of the zoomed in views (squares) of TEM images for different fractions correspond to the colours shown in the bar plot (top left). DOL; DNA origami liposome. Source data for bar plot provided as a Source Data file.

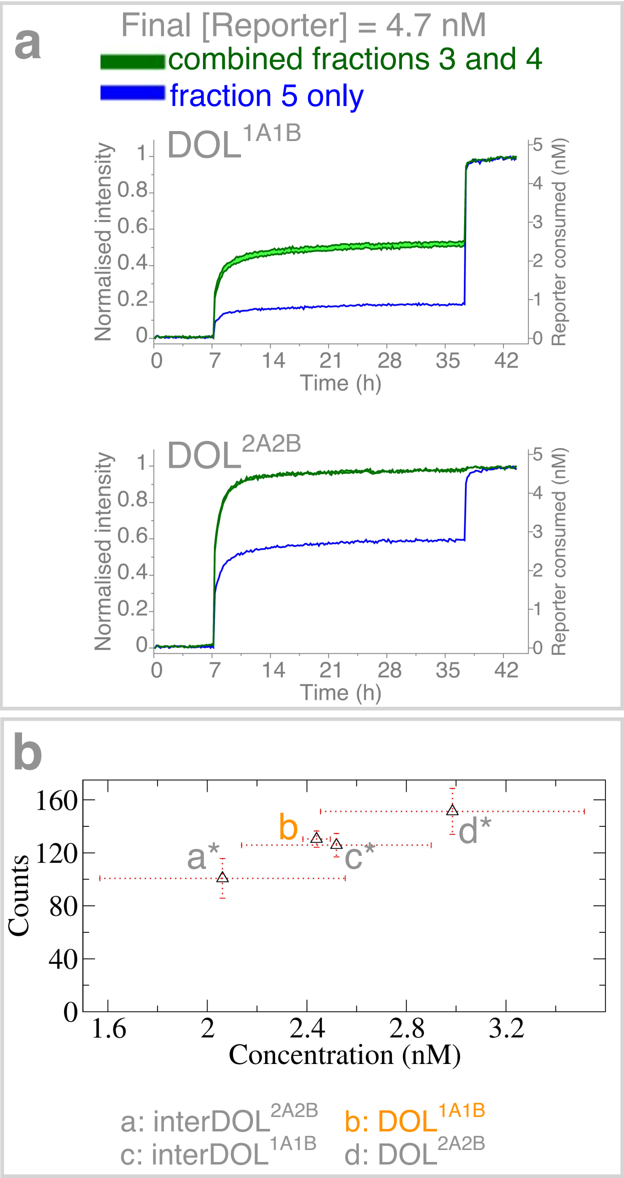

Supplementary Figure 5: Fluorescence for intra-DOL cases and concentration calculation. **(a)** Similar to plate reader experiment in Figure 2d, kinetic curves are shown and compared in each case for pooled 3+4 fraction (two repeats, green curves) vs. fraction 5 (blue curves). **(b)** Counts vs. concentration plot shown, with single standard deviations, for different DOL cases. DOL^{1A1B} TEM data was used to obtain a general relationship between counts and concentration. Linear regression fitting of concentration (from plate reader) of DOL^{1A1B} vs. their averaged TEM counts (4 TEM images from same sample) provided $\text{Concentration}=\frac{\text{Counts}+12}{54.7}$, which was then used to predict concentrations for cases marked with * from their respective TEM data. Calculated concentration of pooled fraction (3+4) for DOL^{2A2B} is 2.98 ± 0.53 nM using TEM data (averaged manual counts from 7 TEM images of pooled fraction '3+4' DOL^{2A2B} same sample). For inter-DOL cases their concentrations were calculated from TEM counts (averaged manual counts from, same sample, 5 TEM images in $interDOL^{1A1B}$ case and from, same sample, 4 TEM images in $interDOL^{2A2B}$ case); 2.52 ± 0.38 nM $interDOL^{1A1B}$ case and 2.06 ± 0.49 nM $interDOL^{2A2B}$ case. Note that interDOL is a mixture of DOLs containing either 1A or 1B, thus concentration of each type of DOL would be approximately half of the calculated concentrations. See Supplementary Note 4. DOL; DNA origami liposome, 1A or 2A; one or two Receptor_A; 1B or 2B, one or two Receptor_B. Source data are provided as a Source Data file.

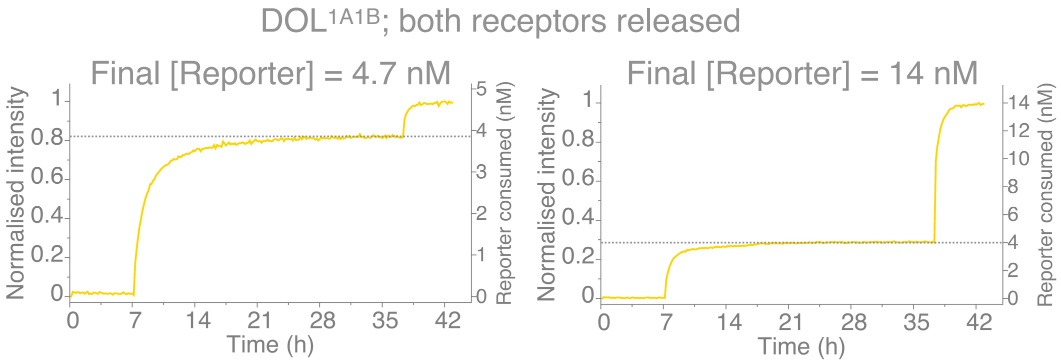

Supplementary Figure 6: Excess reporter complex does not block receptors. Same DOL sample was reacted with two different reporter complex concentrations. In both the cases the saturation end points are marked with dotted horizontal lines. See related discussion in Supplementary Note 3. DOL; DNA origami liposome, 1A; one Receptor_A; 1B, one Receptor_B. Source data are provided as a Source Data file.

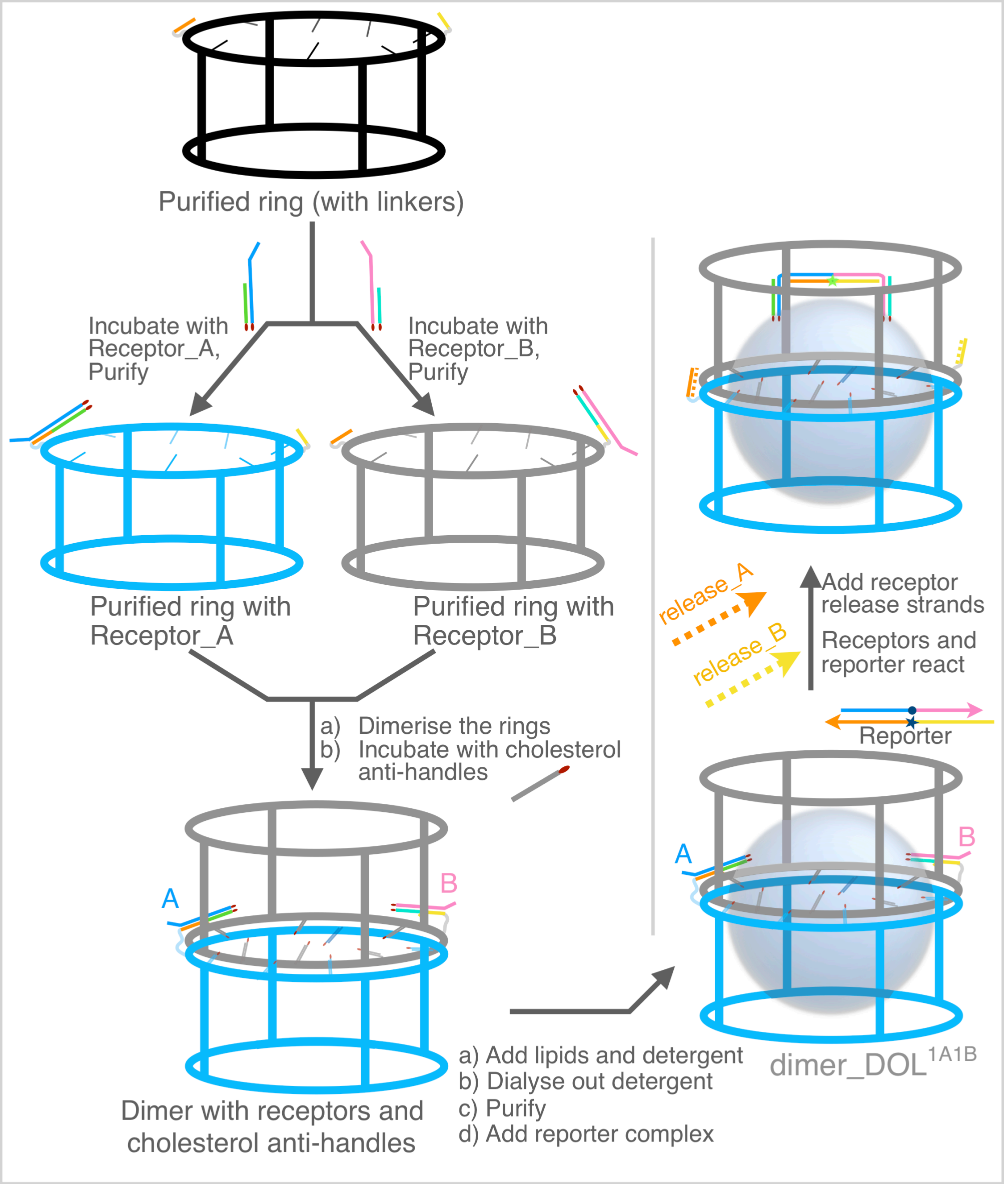

Supplementary Figure 7: Dimer ring platform. Initially each ring was tethered with only one type of receptor and purified. Both the rings were later dimerised with the help of different (thus orientation is maintained) complementary staple extensions from one ring to the scaffold of the other ring. Overall the strategy remains similar to as explained in Figure 1. DOL; DNA origami liposome, A; Receptor_A, B; Receptor_B. DNA domains shown in different colours have a specific role as explained in Supplementary Table 1.

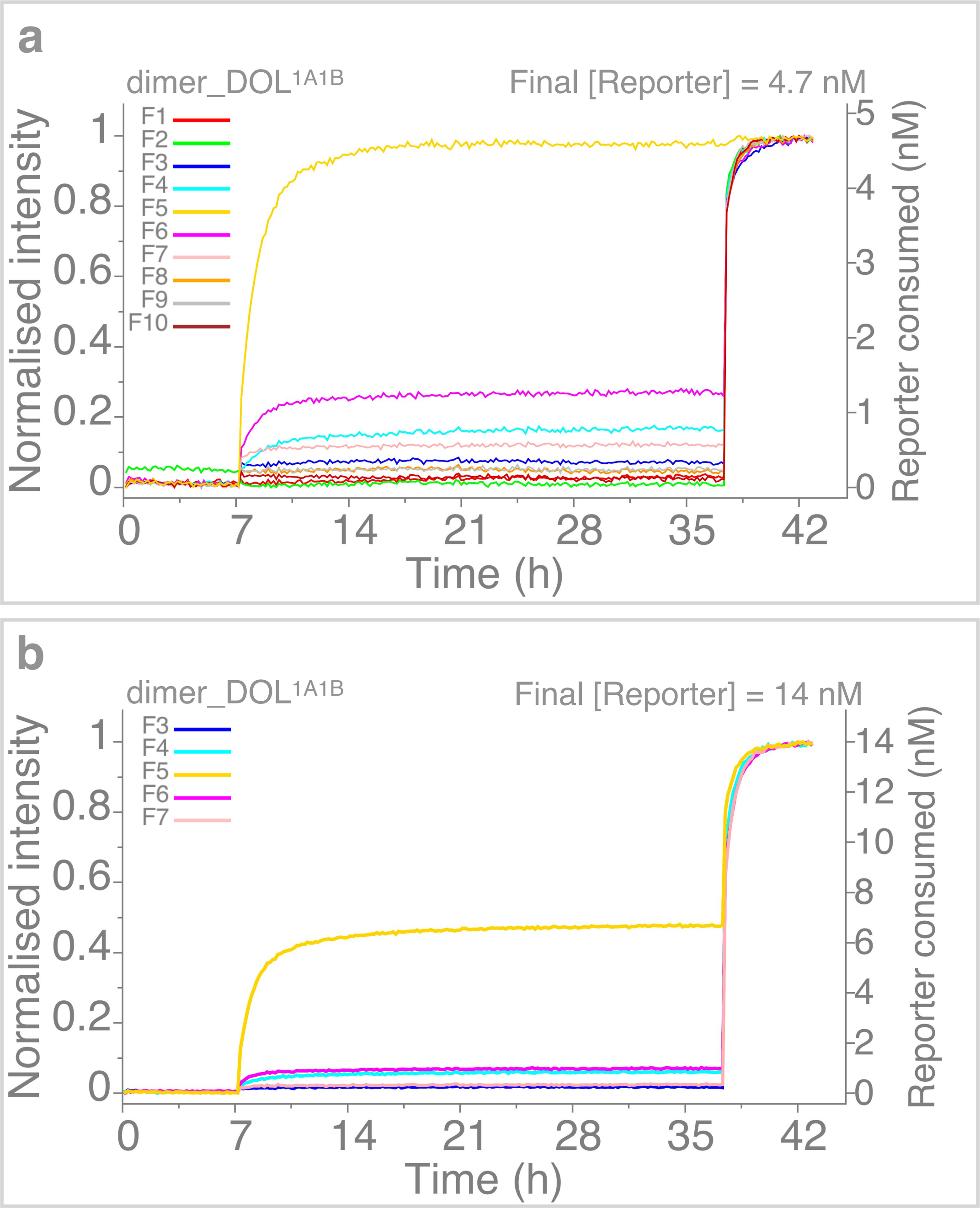

Supplementary Figure 8: Analysing fractions obtained after isopycinic purification for dimer_DOL^{1A1B} case. **(a)** Plate reader experiment showing kinetic curves for fractions 1-10 with reporter concentration 4.7 nM. **(b)** Similar experiment as in **a** using reporter concentration 14 nM with fractions 3-7. Each curve is shown in a different colour corresponds a particular fraction. DOL; DNA origami liposome, 1A; one Receptor_A; 1B, one Receptor_B. Source data are provided as a Source Data file.

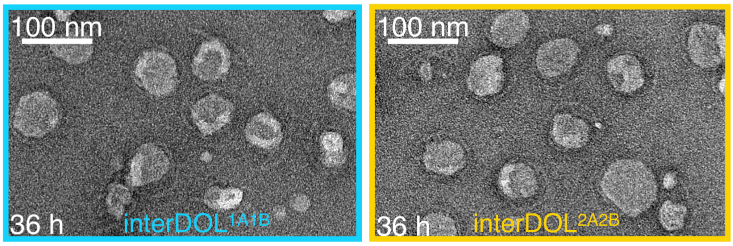

Supplementary Figure 9: TEM images for inter-DOL cases. Samples were taken after the completion of the plate reader experiment shown in Figure 2e. Similar TEM images were obtained in three different experiments (different day and starting materials). DOL; DNA origami liposome, 1A or 2A; one or two Receptor_A; 1B or 2B, one or two Receptor_B.

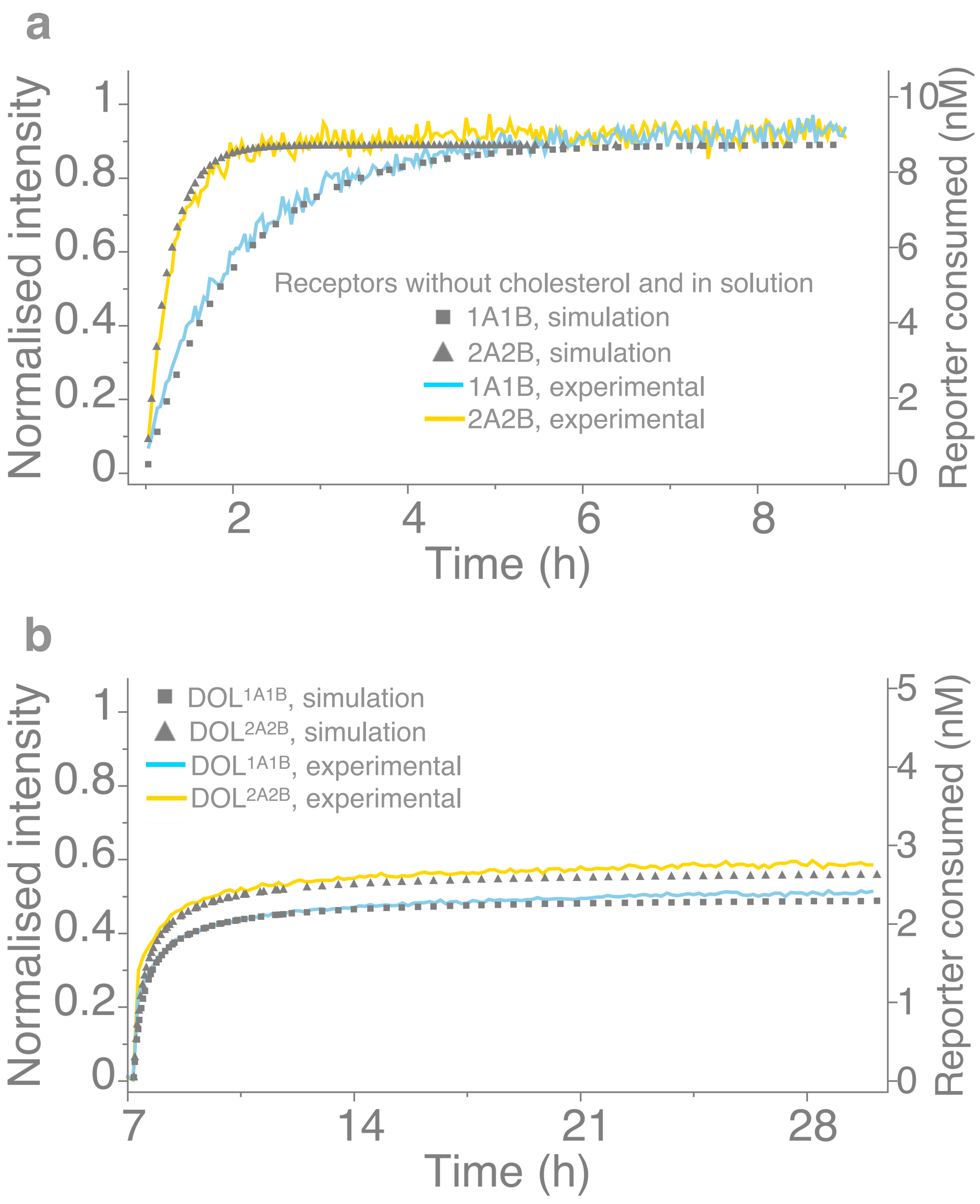

Supplementary Figure 10: Simulation curves. **(a)** Experimental fluorescence kinetics curves in the solution case (Figure 2f) are plotted along with the fitted curves derived from the model discussed in Supplementary Note 6. For curve fitting data points from 1-9 h were used. **(b)** Similar to **a**, experimental (Figure 2d) and fitted curves shown for intra-DOL cases using data points 7-30 h. Blue curves are for 1A1B cases and orange curves are for 2A2B cases (note that 2 in **a** cases means 2x the concentration of receptors compared to 1A1B in solution). DOL; DNA origami liposome, 1A or 2A; one or two Receptor_A; 1B or 2B, one or two Receptor_B. Source data are provided as a Source Data file.

Supplementary References:

(1) Morzy, D.; Rubio-Sánchez, R.; Joshi, H.; Aksimentiev, A.; Di Michele, L.; Keyser, U. F. Cations Regulate Membrane Attachment and Functionality of DNA Nanostructures. *J. Am. Chem. Soc.* **2021**, *143*, 7358–7367.

(2) Stanton, B. Z.; Chory, E. J.; Crabtree, G. R. Chemically Induced Proximity in Biology and Medicine. *Science (80-.).* **2018**, *359*, eaao5902.

(3) David Soloveichik. CRNSimulator Mathematica Package http://users.ece.utexas.edu/~soloveichik/crnsimulator.html.

(4) Srinivas, N.; Ouldridge, T. E.; Šulc, P.; Schaeffer, J. M.; Yurke, B.; Louis, A. A.; Doye, J. P. K.; Winfree, E. On the Biophysics and Kinetics of Toehold-Mediated DNA Strand Displacement. *Nucleic Acids Res.* **2013**, *41*, 10641–10658.

(5) Zhang, D. Y.; Winfree, E. Control of DNA Strand Displacement Kinetics Using Toehold Exchange. *J. Am. Chem. Soc.* **2009**, *131*, 17303–17314.
