# Supplementary material for: Digital nanoreactors to control absolute stoichiometry and spatiotemporal behavior of DNA receptors within lipid bilayers
Source: Nat Commun. 2023 Mar 20;14:1532. doi: 10.1038/s41467-023-36996-x (PMC10027858; doi:10.1038/s41467-023-36996-x)
Supplement: Supplementary file 2 — Description of Additional Supplementary Files [file 41467_2023_36996_MOESM2_ESM.pdf]

Description of Additional Supplementary File

Supplementary Data 1; **Chemicals catalogs**: Single excel file contains catalog numbers of chemicals used in this study.

Supplementary Data 2; **DNA sequences**: All staple DNA sequences for different designs in a single excel file with multiple sheets. 1A1B_sequence sheet has staple sequences for DOL^{1A1B} ring, 2A2B_sequence sheet has staple sequences for DOL^{2A2B} ring, Ring1_for_dimer1A1B_sequence sheet has staple sequences for Ring 1 used in dimer_DOL^{1A1B}, and Ring2_for_dimer1A1B_sequence sheet has staple sequences for Ring 2 used in dimer_DOL^{1A1B}. Ring 1 and Ring 2 together form a full dimer_DOL^{1A1B}.

Supplementary Software: a) cadnano: This folder contains DNA origami design json file which can be used with cadnano2 software. See README_for_caDNAno.txt for more details. b) crn: This folder contains CRN simulator, file CRNSimulator.wl, used in this study (Supplementary Figure 10). Experimental data used for the simulations is provided in xlsx files. 1A1B.xlsx and 2A2B.xlsx are for DOL1A1B and DOL2A2B cases respectively. 1A1B_nonchol.xlsx and 2A2B_nonchol.xlsx are for receptors in the solution cases (each receptor free in solution and without cholesterol modifications). See README file for receptorbinding_final.nb usage. It requires Mathematica 11.2. c) perl_scripts: This folder contains two PERL scripts and sample raw data files which could be used to normalize any data. README file provides the steps on how to use the scripts.
