# Supplementary material for: Digital nanoreactors to control absolute stoichiometry and spatiotemporal behavior of DNA receptors within lipid bilayers
Source: Nat Commun. 2023 Mar 20;14:1532. doi: 10.1038/s41467-023-36996-x (PMC10027858; doi:10.1038/s41467-023-36996-x)

Load data

In[45]:=
```
SetDirectory[NotebookDirectory[]];
```

In[46]:=
```
timeDelay[data_, delay_] :=
 Module[{},
  Table[Thread[{data⟦x, All, 1⟧ + delay, data⟦x, All, 2⟧}], {x, Dimensions[data]⟦1⟧}]
 ]
delay = 20 / 60;
```

In[48]:=
```
solution1A1Bnochol = Import["1A1B_nochol.xlsx"];
solution2A2Bnochol = Import["2A2B_nochol.xlsx"];
oneAoneB = Import["1A1B.xlsx"];
twoAtwoB = Import["2A2B.xlsx"];


oneAoneB = timeDelay[oneAoneB, delay];
twoAtwoB = timeDelay[twoAtwoB, delay];
```

In[54]:=
```
solution1A1Bnocholplot = ListPlot[Tooltip[solution1A1Bnochol⟦1, ;; ;; 2⟧], Frame → True,
  ImageSize → 400, PlotStyle → Table[{PointSize[Medium], i}, {i, {Blue}}],
  PlotRange → {Full, Full}, Joined → False,
  PlotLabel → Style["solution 1A1B", FontFamily → "Helvetica", FontSize → 16, Black],
  PlotLegends → {""},
  FrameLabel → {Style["Time (hours)", Black, FontSize → 16, FontFamily → "Helvetica"],
    Style["Fluorescence", Black, FontSize → 16, FontFamily → "Helvetica"]}]
```

Out[54]=

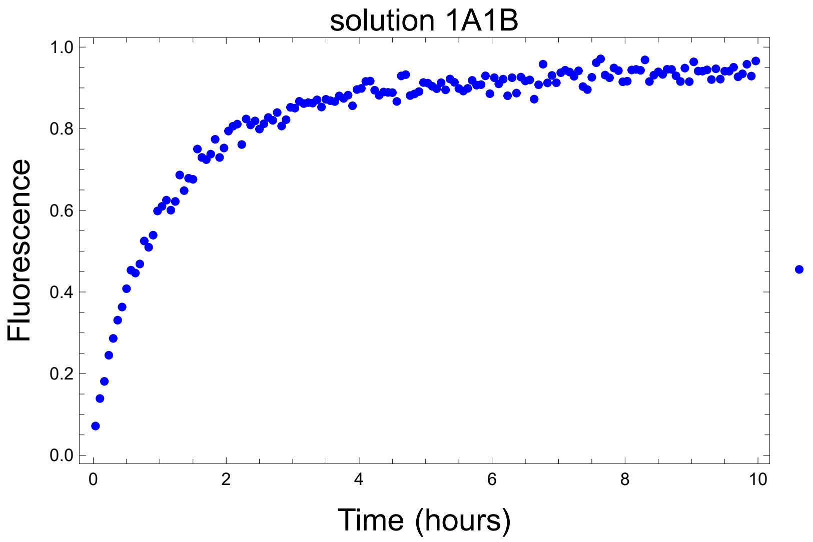

In[55]:=
```
solution2A2Bnocholplot = ListPlot[Tooltip[solution2A2Bnochol[[1, ;; ;; 2]]], Frame → True,
  ImageSize → 400, PlotStyle → Table[{PointSize[Medium], i}, {i, {Green}}],
  PlotRange → {Full, Full}, Joined → False,
  PlotLabel → Style["solution 2A2B", FontFamily → "Helvetica", FontSize → 16, Black],
  PlotLegends → {""},
  FrameLabel → {Style["Time (hours)", Black, FontSize → 16, FontFamily → "Helvetica"],
    Style["Fluorescence", Black, FontSize → 16, FontFamily → "Helvetica"]}]
```

Out[55]=

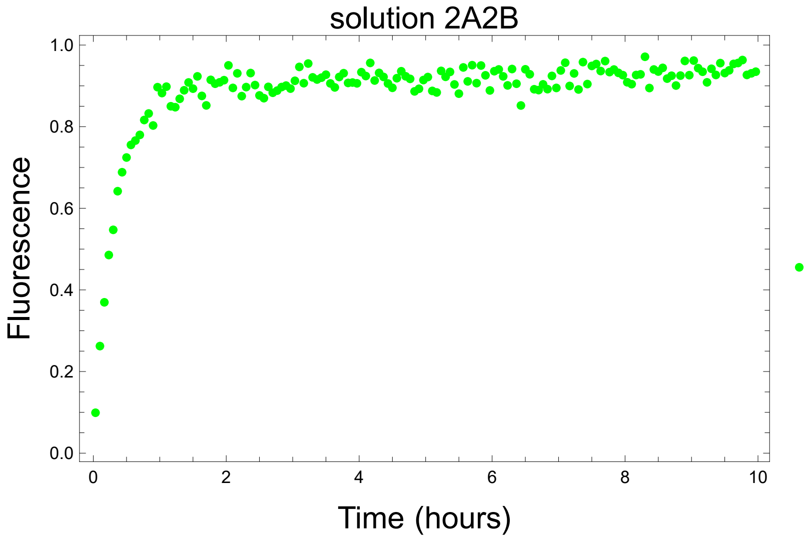

In[56]:=
```
oneA1B = ListPlot[Tooltip[oneAoneB[[1, ;; ;; 2]]], Frame → True,
  ImageSize → 400, PlotStyle → Table[{PointSize[Medium], i}, {i, {Blue}}],
  PlotRange → {Full, Full}, Joined → False,
  PlotLabel → Style["surface 1A1B", FontFamily → "Helvetica", FontSize → 16, Black],
  PlotLegends → {""},
  FrameLabel → {Style["Time (hours)", Black, FontSize → 16, FontFamily → "Helvetica"],
    Style["Fluorescence", Black, FontSize → 16, FontFamily → "Helvetica"]}]
```

Out[56]=

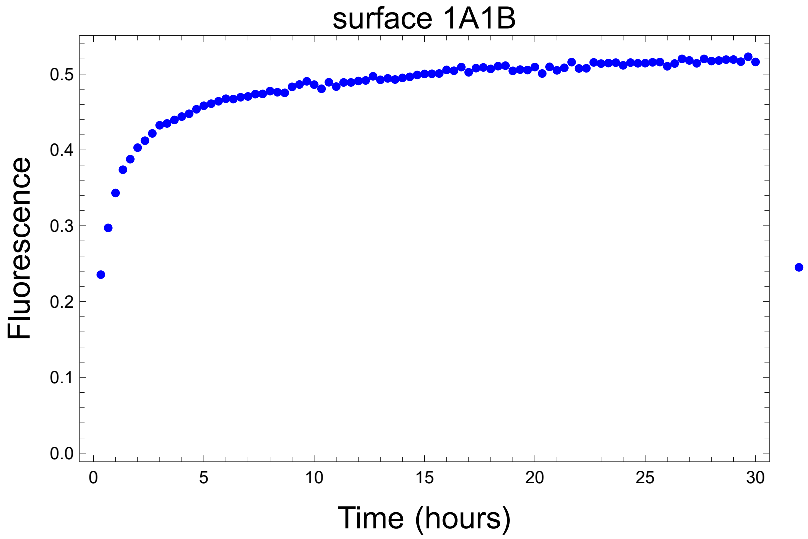

In[57]:=
```
twoA2B = ListPlot[Tooltip[twoAtwoB[[1, ;; ;; 2]]], Frame → True, ImageSize → 400,
  PlotStyle → Table[{PointSize[Medium], i}, {i, {RGBColor["#EACE09"]}}],
  PlotRange → {Full, Full}, Joined → False,
  PlotLabel → Style["surface 2A2B", FontFamily → "Helvetica", FontSize → 16, Black],
  PlotLegends → {""},
  FrameLabel → {Style["Time (hours)", Black, FontSize → 16, FontFamily → "Helvetica"],
    Style["Fluorescence", Black, FontSize → 16, FontFamily → "Helvetica"]}]
```

Out[57]=

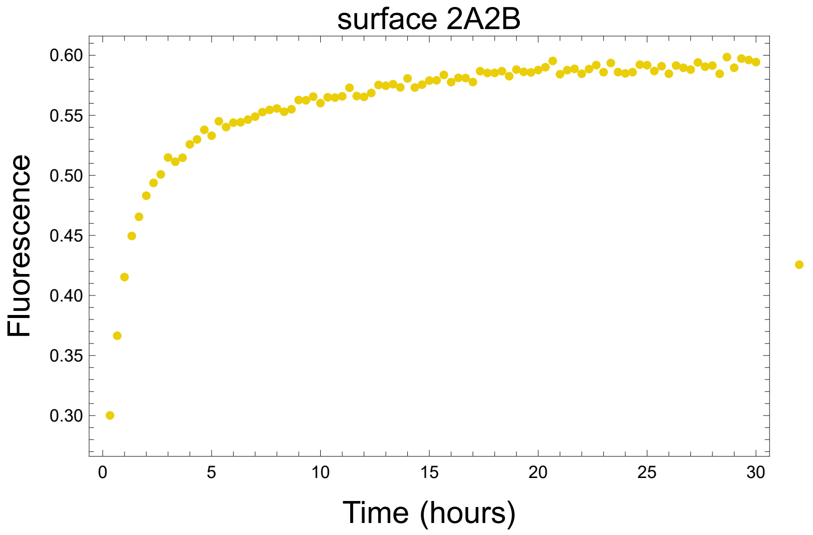

Simulations

In[58]:=
```
SetDirectory[NotebookDirectory[]];
```

In[59]:=
```
Get["CRNSimulator.wl"];
```

In[60]:=
```
Conc = 10^-9; (* unit: M *)
RelConc = 95 * 10^-9 ;(* unit: M *)
SimulationTime = 24; (* unit: hours *)
keff = 2 * 10^5;(* unit: /M/s *)
keff2 = 7.6 * 10^4; (* unit: /M/s *)
kf = 2 * 10^6;
(* unit: /M/s *)
kb = 10;(* dissociation rate; unit: /s *)
```

In[67]:=
```
solconcs = {{900, 900, 100, 100, 1, 11}, {1800, 1800, 200, 200, 1, 11}};
(*concentrations of release strand A, release strand B,
receptor A, and receptor B for 1A1B and 2A2B without cholesterol*)
```

In[68]:=
```
ReceptorBindingNoChol[keff_, keff2_] := Table[
   sol = SimulateRxnsys[
     {rxn[releaseA + receptorA, freeReceptorA + waste, keff],
      rxn[releaseB + receptorB, freeReceptorB + waste, keff],
      revrxn[freeReceptorA + Rep, freeReceptorARep, kf, kb],
      revrxn[freeReceptorB + Rep, freeReceptorBRep, kf, kb],
      rxn[freeReceptorARep + freeReceptorB, Tcomp + waste, i[[5]] * keff2],
      rxn[freeReceptorBRep + freeReceptorA, Tcomp + waste, i[[5]] * keff2],
      conc[releaseA, i[[1]] * Conc],
      conc[releaseB, i[[2]] * Conc],
      conc[receptorA, i[[3]] * Conc],
      conc[receptorB, i[[4]] * Conc],
      conc[Rep, 9.8 * 10^-9]
     },
     SimulationTime * 60 * 60
    ];
   Tcomp[t * 60 * 60] / (i[[6]] * Conc) /. sol,
   {i, solconcs}];
```

```
In[69]:= ReceptorBindingNoCholKinetics4hr[keff_, keff2_] :=
  Plot[Evaluate[ReceptorBindingNoChol[keff, keff2]], {t, 0, SimulationTime},
   Frame → True, GridLines → Automatic,
   FrameLabel → {Style["Time (hrs)", FontSize → 20, FontFamily → "Helvetia"],
     Style["reporter triggered",
      FontSize → 20, FontFamily → "Helvetia", LineSpacing → {0.2, 0}]},
   PlotStyle → Table[{AbsoluteThickness[2], i}, {i, {Blue, Green, Darker[Green]}}],
   PlotLabel → Style["no chol data, keff2=7.6*10^4/M/s, 4 hr",
     FontFamily → "Helvetica", FontSize → 16, Black],
 PlotLegends → SwatchLegend[Automatic, {"1A1B", "2A2B"},
     LegendLabel → Row[{""}],
     LabelStyle → Directive[FontSize → 20, FontFamily → "Helvetia"],
     LegendMarkerSize → 14],
   LabelStyle → Directive[FontSize → 20, FontFamily → "Helvetia"],
 PlotRange → {{0, 4}, {-0.1, 1.15}}, ImageSize → 400, AspectRatio → 1 / 1.5]

 ReceptorBindingNoCholKineticsAll[keff_, keff2_] :=
  Plot[Evaluate[ReceptorBindingNoChol[keff, keff2]], {t, 0, SimulationTime},
   Frame → True, GridLines → Automatic,
   FrameLabel → {Style["Time (hrs)", FontSize → 20, FontFamily → "Helvetia"],
     Style["reporter triggered",
      FontSize → 20, FontFamily → "Helvetia", LineSpacing → {0.2, 0}]},
   PlotStyle → Table[{AbsoluteThickness[2], i}, {i, {Blue, Green, Darker[Green]}}],
   PlotLabel → Style["no chol data, keff2=7.6*10^4/M/s, 10 hr",
     FontFamily → "Helvetica", FontSize → 16, Black],
 PlotLegends → SwatchLegend[Automatic, {"1A1B", "2A2B", "DOLr1 1A1B"},
     LegendLabel → Row[{""}],
     LabelStyle → Directive[FontSize → 20, FontFamily → "Helvetia"],
     LegendMarkerSize → 14],
   LabelStyle → Directive[FontSize → 20, FontFamily → "Helvetia"],
 PlotRange → {{0, 10}, {-0.1, 1.15}}, ImageSize → 400, AspectRatio → 1 / 1.5]
```

In[71]:=
```
Show[ReceptorBindingNoCholKinetics4hr[2 * 10^5, 7.6 * 10^4],
  solution1A1Bnocholplot, solution2A2Bnocholplot]
```

Out[71]=

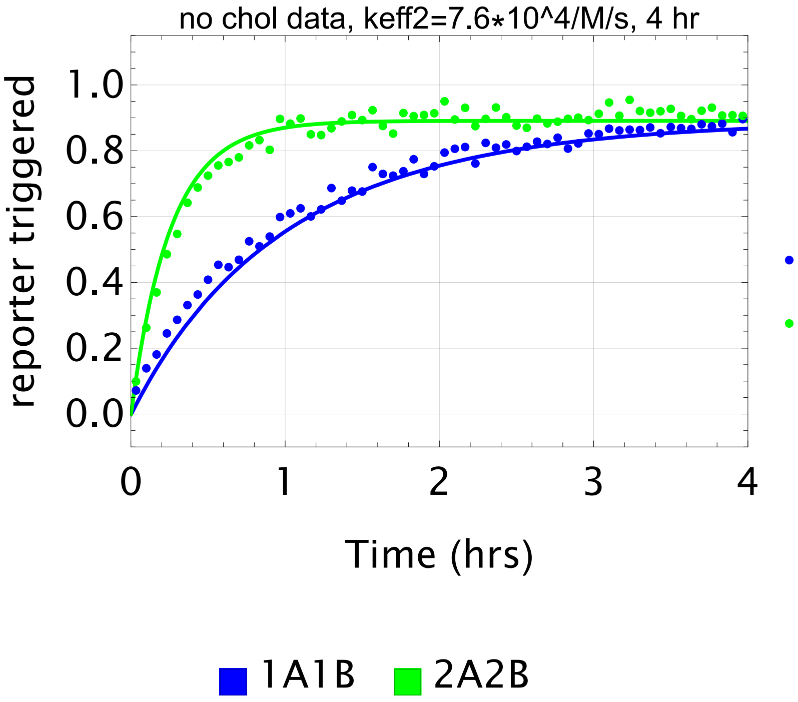

In[72]:=

In[73]:=
```
Show[ReceptorBindingNoCholKineticsAll[2 * 10^5, 7.6 * 10^4],
 solution1A1Bnocholplot, solution2A2Bnocholplot]
```

Out[73]=

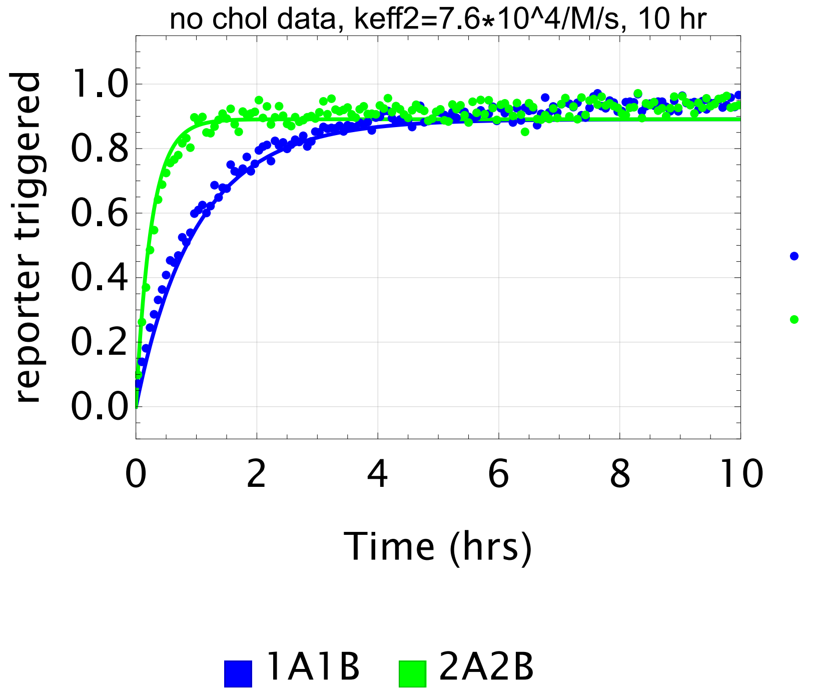

In[74]:=
```
concentrations = {{2.35, 2.35}, {1.35 * 2, 1.35 * 2}};
(*concentrations of receptor A and receptor B for 1A1B and 2A2B*)
```

In[75]:=
```
ReceptorBinding[] := Table[
   sol = SimulateRxnsys[
     {rxn[releaseA + receptorA, freeReceptorA + waste, keff],
      rxn[releaseB + receptorB, freeReceptorB + waste, keff],
      revrxn[freeReceptorA + Rep, freeReceptorARep, 2 * 10^6, 10],
      revrxn[freeReceptorB + Rep, freeReceptorBRep, 2 * 10^6, 10],
      rxn[freeReceptorARep + freeReceptorB, Tcomp + waste, 2800 * keff2],
      rxn[freeReceptorBRep + freeReceptorA, Tcomp + waste, 2800 * keff2],
      conc[releaseA, RelConc],
      conc[releaseB, RelConc],
      conc[receptorA, i[[1]] * Conc],
      conc[receptorB, i[[2]] * Conc],
      conc[Rep, 4.7 * 10^-9]
     },
     SimulationTime * 60 * 60
    ];
   Tcomp[t * 60 * 60] / (4.7 * Conc) /. sol,
   {i, concentrations}];
```

In[76]:=

In[77]:=
```
ReceptorBindingKinetics4hr[] := Plot[Evaluate[ReceptorBinding[]], {t, 0, SimulationTime},
  Frame → True, GridLines → Automatic,
  FrameLabel → {Style["Time (hrs)", FontSize → 20, FontFamily → "Helvetia"],
    Style["reporter triggered",
     FontSize → 20, FontFamily → "Helvetia", LineSpacing → {0.2, 0}]},
  PlotStyle → Table[{AbsoluteThickness[2], i}, {i, {Blue, Yellow}}],
  PlotLabel → Style["1A1B and 2A2B", FontFamily → "Helvetica", FontSize → 16, Black],
PlotLegends → SwatchLegend[{"1A1B,1x=2.35nM", "2A2B,1x=1.35nM"},
    LegendLabel → Row[{""}],
    LabelStyle → Directive[FontSize → 20, FontFamily → "Helvetia"],
    LegendMarkerSize → 14],
  LabelStyle → Directive[FontSize → 20, FontFamily → "Helvetia"],
PlotRange → {{0, 4}, {-0.1, 1.15}}, ImageSize → 400, AspectRatio → 1 / 1.5]
```

In[78]:=
```
Show[ReceptorBindingKinetics4hr[], oneA1B, twoA2B]
```

Out[78]=

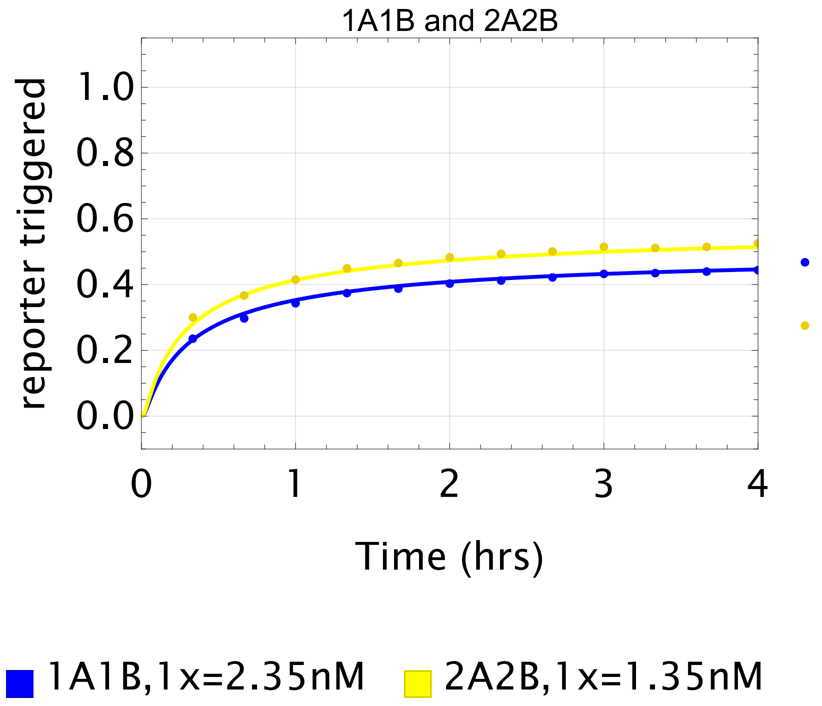

In[79]:=
```
ReceptorBindingKinetics24hr[] :=
 Plot[Evaluate[ReceptorBinding[]], {t, 0, SimulationTime},
  Frame → True, GridLines → Automatic,
  FrameLabel → {Style["Time (hrs)", FontSize → 20, FontFamily → "Helvetia"],
    Style["reporter triggered",
     FontSize → 20, FontFamily → "Helvetia", LineSpacing → {0.2, 0}]},
  PlotStyle → Table[{AbsoluteThickness[2], i}, {i, {Blue, Yellow}}],
  PlotLabel → Style["1A1B and 2A2B", FontFamily → "Helvetica", FontSize → 16, Black],
PlotLegends → SwatchLegend[Automatic, {"1A1B,1x=2.35nM", "2A2B,1x=1.35nM"},
    LegendLabel → Row[{""}],
    LabelStyle → Directive[FontSize → 20, FontFamily → "Helvetia"],
    LegendMarkerSize → 14],
  LabelStyle → Directive[FontSize → 20, FontFamily → "Helvetia"],
PlotRange → {{0, 24}, {-0.1, 1.15}}, ImageSize → 400, AspectRatio → 1 / 1.5]
```

In[80]:=
```
Show[ReceptorBindingKinetics24hr[], oneA1B, twoA2B]
```

Out[80]=

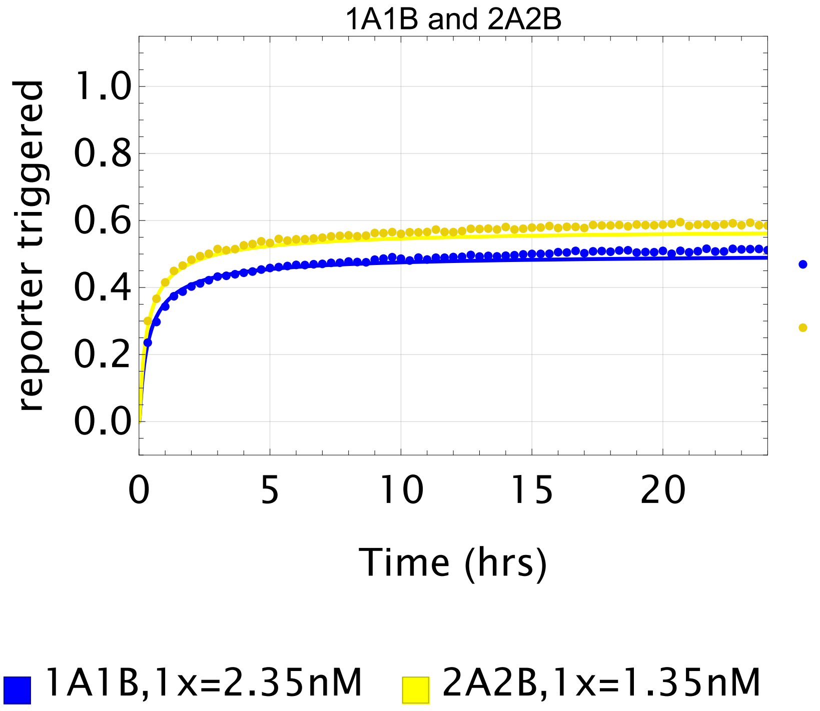

Supplement: Supplementary file 5 — Supplementary Software [file 41467_2023_36996_MOESM5_ESM.zip › Supplementary_Software/crn/receptorbinding_final.pdf]
