# Supplementary material for: Digital nanoreactors to control absolute stoichiometry and spatiotemporal behavior of DNA receptors within lipid bilayers
Source: Nat Commun. 2023 Mar 20;14:1532. doi: 10.1038/s41467-023-36996-x (PMC10027858; doi:10.1038/s41467-023-36996-x)
Supplement: Supplementary file 6 — Reporting Summary [file 41467_2023_36996_MOESM6_ESM.pdf]

nature portfolio

Corresponding author(s): Vishal Maingi
Chris Thachuk
Edwin R Chapman
Paul W K Rothemund

Last updated by author(s): Feb 14, 2023

Reporting Summary

Nature Portfolio wishes to improve the reproducibility of the work that we publish. This form provides structure for consistency and transparency in reporting. For further information on Nature Portfolio policies, see our Editorial Policies and the Editorial Policy Checklist.

Statistics

For all statistical analyses, confirm that the following items are present in the figure legend, table legend, main text, or Methods section.

n/a	Confirmed	
☐	☒	The exact sample size (n) for each experimental group/condition, given as a discrete number and unit of measurement
☐	☒	A statement on whether measurements were taken from distinct samples or whether the same sample was measured repeatedly
☒	☐	The statistical test(s) used AND whether they are one- or two-sided<br>*Only common tests should be described solely by name; describe more complex techniques in the Methods section.*
☒	☐	A description of all covariates tested
☐	☒	A description of any assumptions or corrections, such as tests of normality and adjustment for multiple comparisons
☐	☒	A full description of the statistical parameters including central tendency (e.g. means) or other basic estimates (e.g. regression coefficient) AND variation (e.g. standard deviation) or associated estimates of uncertainty (e.g. confidence intervals)
☒	☐	For null hypothesis testing, the test statistic (e.g. F, t, r) with confidence intervals, effect sizes, degrees of freedom and P value noted<br>*Give P values as exact values whenever suitable.*
☒	☐	For Bayesian analysis, information on the choice of priors and Markov chain Monte Carlo settings
☒	☐	For hierarchical and complex designs, identification of the appropriate level for tests and full reporting of outcomes
☒	☐	Estimates of effect sizes (e.g. Cohen's d, Pearson's r), indicating how they were calculated

Our web collection on statistics for biologists contains articles on many of the points above.

Software and code

Policy information about availability of computer code

Data collection	cadnano2 for designing DNA rings and staple sequences, Mathematica11.2 and CRN simulator 2012 (provided as Supplementary Software) for kinetics simulations plot
Data analysis	Mathematica11.2 for kinetics simulations analyses, PERL v5.16.3 for normalizing data, gnuplot v5.4 and xmgrace Grace-5.1 for plotting graphs.

For manuscripts utilizing custom algorithms or software that are central to the research but not yet described in published literature, software must be made available to editors and reviewers. We strongly encourage code deposition in a community repository (e.g. GitHub). See the Nature Portfolio guidelines for submitting code & software for further information.

Data

Policy information about availability of data

All manuscripts must include a data availability statement. This statement should provide the following information, where applicable:

- Accession codes, unique identifiers, or web links for publicly available datasets
- A description of any restrictions on data availability
- For clinical datasets or third party data, please ensure that the statement adheres to our policy

Data in Figures 2 and 3, Supplementary Figures 3, 4, 5, 6, 8, and 10, and sequences in Table 1 and Supplementary Table 1 are provided in the Source Data file. Source Data are provided with this paper. Chemicals catalogs are provided as Supplementary Data 1 and DNA staple sequences are provided as Supplementary Data

2 files.

Human research participants

Policy information about studies involving human research participants and Sex and Gender in Research.

Reporting on sex and gender	Information not collected (not required for our study)
Population characteristics	Information not collected (not required for our study)
Recruitment	Not required for our study
Ethics oversight	Not required for our study

Note that full information on the approval of the study protocol must also be provided in the manuscript.

Field-specific reporting

Please select the one below that is the best fit for your research. If you are not sure, read the appropriate sections before making your selection.

[x] Life sciences [ ] Behavioural & social sciences [ ] Ecological, evolutionary & environmental sciences

For a reference copy of the document with all sections, see nature.com/documents/nr-reporting-summary-flat.pdf

Life sciences study design

All studies must disclose on these points even when the disclosure is negative.

Sample size	Sample size is mentioned in the study and the standard deviations are provided. No sample-size calculation was performed. We follow common practice in the field, where each experiment was repeated 2-3 times on different occasions (different day, different starting sample) with 2-3 copies for many samples on each occasion. Larger sample size is prevented by practical limitations (sample quantity). Nevertheless, data are highly reproducible in this in vitro setup, and results from different experiments corroborate each other.
Data exclusions	No data excluded
Replication	Each plate reader experiment was repeated 2-3 times (using different batches of samples on different day) with 2-3 copies for many samples on each occasion. Each TEM experiment was repeated 2-3 times (using different batches of samples on different day) with 3-10 images of each sample. The data for each sample are consistent.
Randomization	Plate reader well positions were chosen randomly and data was normalized with respect to each well independently (baseline fluorescence subtracted). Other allocation randomization is not relevant in this study.
Blinding	The person who performed TEM imaging was blinded to the sample identities. No blinding was performed in plate reader experiments because the final kinetics analysis is quantitative which does not require individual judgment or interpretation of the data.

Reporting for specific materials, systems and methods

We require information from authors about some types of materials, experimental systems and methods used in many studies. Here, indicate whether each material, system or method listed is relevant to your study. If you are not sure if a list item applies to your research, read the appropriate section before selecting a response.

Materials & experimental systems

n/a	Involved in the study	
[x]	[ ]	Antibodies
[x]	[ ]	Eukaryotic cell lines
[x]	[ ]	Palaeontology and archaeology
[x]	[ ]	Animals and other organisms
[x]	[ ]	Clinical data
[x]	[ ]	Dual use research of concern

Methods

n/a	Involved in the study	
[x]	[ ]	ChIP-seq
[x]	[ ]	Flow cytometry
[x]	[ ]	MRI-based neuroimaging
